# Insight into the preparation of the 2016 *M*_S_6.4 Menyuan earthquake from terrestrial gravimetry-derived crustal density changes

**DOI:** 10.1038/s41598-019-54581-5

**Published:** 2019-12-03

**Authors:** Songbai Xuan, Shuanggen Jin, Yong Chen, Jiapei Wang

**Affiliations:** 1 School of Remote Sensing and Geomatics Engineering, Nanjing University of Information Science and Technology, Nanjing, 210044 China; 2 Nanjing Longyuan Microelectronic Co., LTD, Nanjing, 211106 China; 3 Institute of Seismology, China Earthquake Administration, Wuhan, 430071 China

**Keywords:** Geodynamics, Geophysics, Seismology

## Abstract

Geophysical processes of the pre-earthquake activities are difficult to be determined since less pre-seismic signal is observed directly. Crustal density changes derived from the periodical terrestrial gravimetry may provide meaningful deep information for the pre-earthquake cue. In this study, the crustal density changes following the 2016 *M*_S_6.4 Menyuan earthquake are estimated using ground-based gravity-change data from 2011 to 2015 in the northeastern Tibetan Plateau. The results show that negative density changes dominate the region between the South Longshou Mountain fault and the Daban Mountain fault except the southeast of this region (the seismic region) during 2011–2012. Positive density changes appeared in the middle crust near the epicenter during 2012–2013 and in the upper and middle crust east of the epicenter approximately 1.5 years before the earthquake (2013–2014), and then negative density changes appeared under and northeast of the epicenter approximately four months before the earthquake (2014–2015). The state of the crustal materials near the seismic region changed from convergence to expansion, in turn, indicating that the characteristics of the deep seismogenic process was corresponding to Amos Nur’s 1974 dilatancy-fluid diffusion model.

## Introduction

Temporal–spatial gravity changes from terrestrial gravimetry have contributed to understanding of the tectonic movements including earthquakes^1,2^. Relationship between gravity changes and earthquakes was first reported following the 1964 Alaska earthquake^3^, and then had been attracted much attention. For decades, terrestrial gravity-change data were remarkably employed to investigate the geophysical processes of the earthquakes^4–12^. Accordingly, the models related to the earthquake-induced gravity changes, such as the dilatancy mode^7–9^ and the dislocation mode^13–15^, were proposed to interpret or formulate the earthquake-related deep processes. On account of the unreachability, several remaining questions, such as the seismogenic process, require more available geophysical and geodetic observations on the earth’s surface, as well as the available interpreting methods.

The main factor contributing to gravity changes should be crustal mass redistribution, which generally appears in the forms of the crustal deformation and density changes (DCs)^2,5,16,17^. The surface vertical deformation and rock density changes are regarded as the most significant patterns for local crustal movement^17^. Geodetic observation can intuitively present the surface vertical deformation^18,19^, while the crustal DCs could not be determined directly. Investigating the crustal DCs should be a starting point to understand the seismogenic process. As a tentative research, Xuan *et al*.^20^ suggested that the crustal DCs derived from the temporal-spatial gravity-change data may imply the dynamic information related to the pre-earthquake.

The 2016 *M*_S_6.4 Menyuan earthquake ruptured on the Lenglongling (LLL) fault, only 10 km from the largest event since the 26 August 1986 Menyuan *M*_S_6.4 earthquake. The epicenter locates at the northeastern Tibetan Plateau (NETP), between the east end of the North Qilian Mountain (NQM) fault and the west end of the Tertiary Menyuan basin. The LLL fault, the western segment of the Haiyuan fault, is a left-lateral strike-slip fault with a small dip-slip component initiating in the late Quaternary, and its late Pleistocene slip rate is estimated at 3–24 mm/yr^21–24^. The LLL fault is subjected to an almost NEE-trending compression resulting from the interaction between the Tibetan Plateau and the Alxa block^24,25^. Notably, the focal mechanism and source parameters of the 2016 event^26^ indicated a thrust-type earthquake which is in disaccord with the behavior of the fault^19^.

The observed gravity changed from increase to decrease near the LLL fault and surroundings before the 2016 Menyuan *M*_S_6.4 earthquake^27–29^, suggesting the dynamic information related to the deep process. In this study, the gravity changes following the Menyuan earthquake with time on a one-year scale in the NETP were used to determine the crustal DCs using 3D gravity inversion method. Based on the DC results, the change characteristics of crustal materials are investigated before this event as well as possible factors contributing to the earthquake.

## Results

### Statistical information of the inversion results

**Figure 1:**
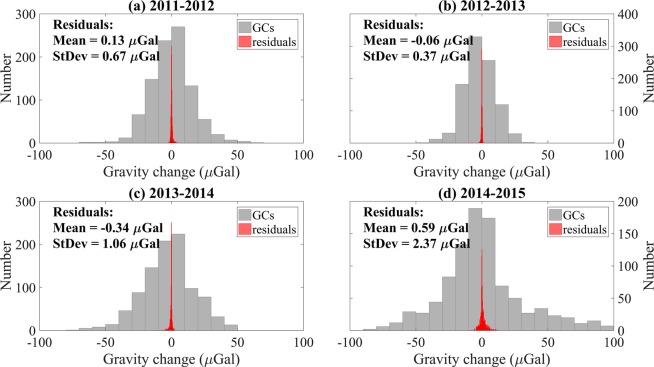
Gravity changes (GCs) and residuals of the inversion results with statistical data. Width of each rectangle on the X-axis (gravity axis) indicates 10 and 0.25 μGal for GCs and residuals, respectively.

**Figure 2:**
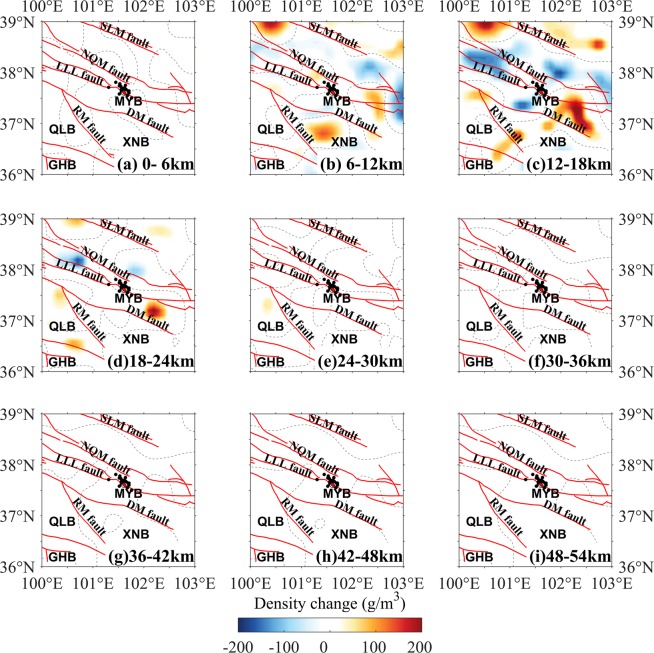
Layered map of the density change in the period 2011–2012. The dotted lines are density change of 0 g/m^3^. The red lines are tectonic faults. The focal mechanism solutions of the 2016 Menyuan earthquake are from USGS^26^. The black dots indicate the aftershocks of the 2016 Menyuan earthquake. *MYB*, Menyuan basin; *XNB*, Xining basin; *QLB*, Qinghai Lake basin; *GHB*, Gonghe basin; *SLM* fault, South Longshou Mountains fault; *NQM* fault, North Qilian Mountains fault; *LLL* fault, Lenglongling fault; *DM* fault, Daban Mountains fault; *RM* fault, Riyue Mountains fault.

**Figure 3:**
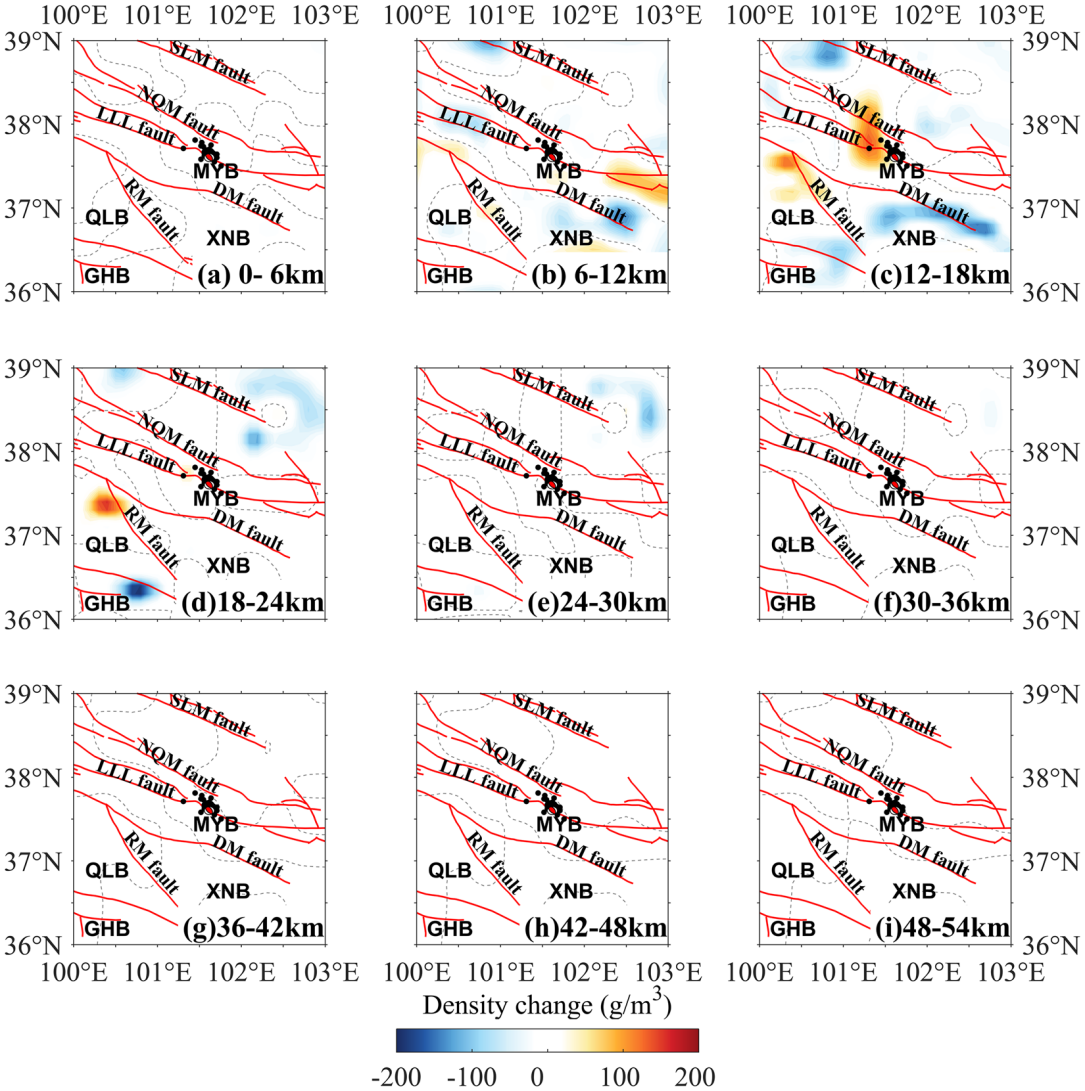
Layered map of the density change in the period 2012–2013. The focal mechanism solutions of the 2016 Menyuan earthquake are from USGS^26^. The dotted lines are density change of 0 g/m^3^. The descriptions are the same as those shown in Fig. 2.

**Figure 4:**
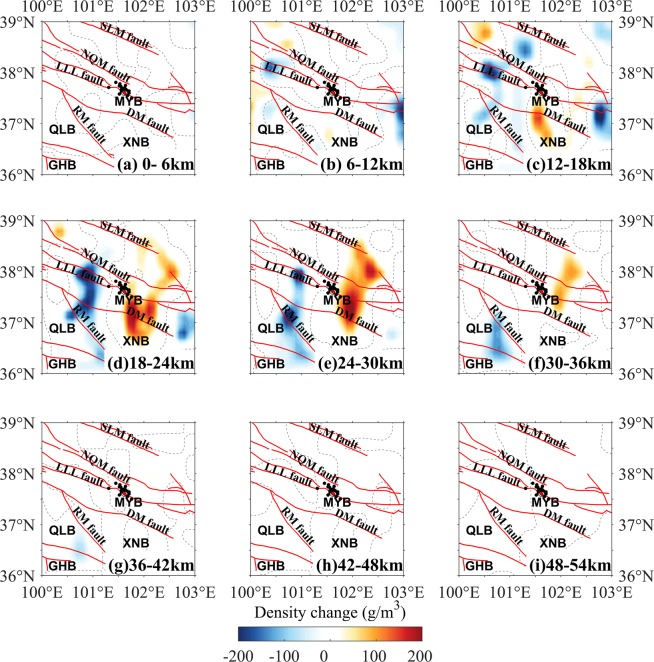
Layered map of the density change in the period 2013–2014. The focal mechanism solutions of the 2016 Menyuan earthquake are from USGS^26^. The dotted lines are density change of 0 g/m^3^. The descriptions are the same as those shown in Fig. 2.

**Figure 5:**
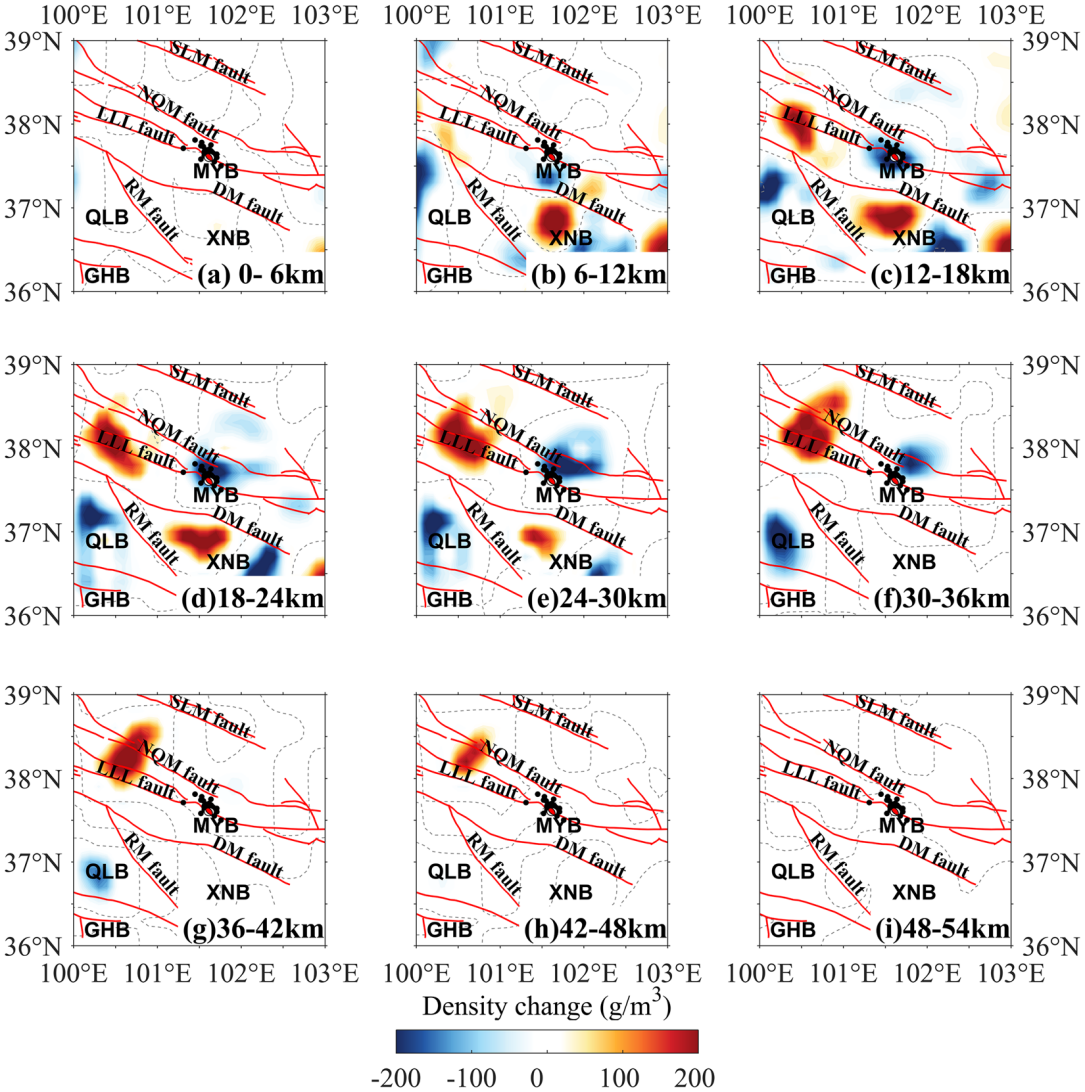
Layered map of the density change in the period 2014–2015. The focal mechanism solutions of the 2016 Menyuan earthquake are from USGS^26^. The dotted lines are density change of 0 g/m^3^. The descriptions are the same as those shown in Fig. 2.

The underground domain, from the surface to a depth of 54 km, included nine layers with non-density-change and had been taken as the initial target model. In the model this domain was divided into 8649 prisms of dimensions 11.1 km (east–west direction) by 11.1 km (north–south direction) by 6 km (vertical direction). During the inversion, the densities of the crustal rock changed within ± 0.2 kg/m^3^. When the standard deviation was less than 5 μGals (1 μGal = 10^−8^ m/s^2^) or after ten steps, the iteration was stopped. Figure 1 presented the gravity-change results and statistics including the residuals; the averages of the residuals range from −0.06 to 0.59 μGal, and the standard deviation (StDev in Fig. 1) varied from 0.37 to 2.37 μGal, confirming the credibility of the inversion results. The layered DC maps were shown in Figs. 2–5.

### Crustal DCs during 2011–2012

Figure 2 showed the layered crustal DCs during the period 2011–2012. The strong DCs were mostly in the upper and middle crust, at depths from 6 km to 24 km (Fig. 2b–d). In the 6–12-km layer (Fig. 2b), positive DCs were found beneath the Xining Basin outlined by the Riyue Mountain (RM) fault and the Daban Mountain (DM) fault, but not at other depths. The negative DCs between the LLL fault and the North Qilian Mountain (NQM) could be clearly found in the 12–18-km layer beneath the northwestern region of the study area (Fig. 2c) and in the 18–24-km layer (Fig. 2d). In contrast, negative DCs were presented in the southeastern region of the study area (Fig. 2c). The anomalous bodies with negative DCs distributed in the region between the South Longshou Mountain (SLM) and the DM faults, excluding the southeastern region between the DM and NQM faults, where showed the positive DCs (Fig. 2c). This anomalous body with positive DCs extended down to a depth of 24 km (Fig. 2d). The distribution of the positive and negative DCs suggested that the expansion regions located in the northeast, northwest and south of the epicenter; however, the convergence region located near the southeast segment of the LLL fault.

### Crustal DCs during 2012–2013

The DCs during 2012–2013 (Fig. 3) distributed also mainly in the upper and middle crust in the northwest region between the LLL fault and the NQM fault. Compared with the DCs during the period of 2011–2012 (Fig. 2), the density remains negative, and beneath the east segment of the LLL fault the density remained positive but was weaker in the 6–12-km layer (Fig. 3b). In the 12–18-km layer (Fig. 3c), a body with positive DCs existed west of the epicenter across the LLL fault, and a negative DC body extended roughly along the east segment of the DM fault, suggesting the middle crustal materials converged to the west of the epicenter of the 2016 Menyuan earthquake, which perhaps was a forewarning phenomenon for the earthquake.

### Crustal DCs during 2013–2015

The crustal DCs near the epicenter of the 2016 Menyuan earthquake during the periods of 2013–2014 and 2014–2015 (Figs. 4 and 5) were of great interest. In 2013–2014, the most significant anomalous DCs were positive and existed at 18–24-km, 24–30-km and 30–36-km layers, indicating convergence of the middle crustal materials into the region east of the epicenter which formed a significant anomalous body. The north margin of this anomalous body with positive DCs roughly aligned with the SLM fault, suggesting that the SLM fault was the north margin of the extrusion of the Tibetan Plateau. In the west region of the study area, negative DCs appeared at depths of 6–42 km (Fig. 4b–g). A distinct north–south trending anomalous belt of negative DCs extending from the LLL fault to the RM fault was found approximately 100 km west of the epicenter at 12–18-km, 18–24-km and 24–30-km layers (Fig. 4d–f), and gradually sloped from south to north with depth, which suggested an eastward expansion of the upper and middle crustal materials.

Most of the intense DCs occurred before the 2016 Menyuan earthquake in the 2014–2015 analysis map (Fig. 5). Among these, three anomalous bodies around the epicenter were of interest. The first body with positive DCs located between the DM and the NQM faults west of the study area. This anomalous body appeared from the middle crust (12–18-km layer) down to the lower crust (45-km layer). However, this region was dominated by negative density changes from 2011–2012 (Fig. 2) to 2013–2014 (Fig. 4); consequently, the DCs was from negative to positive suggested that the state of the crustal materials transformed from expansion to convergence. The second anomalous body was also a positive DC body beneath the Xining Basin at depths of 6–30 km (Fig. 5b–e). Positive DCs also appeared in this region in 2011–2012, but only in the 6–12-km layer (Fig. 2b), which suggested the convergence of the upper and middle crustal materials in this region. The third anomalous body was near the epicenter with negative density changes at depths of 6–36 km (Fig. 5b–f) and with dipping from southwest to northeast with depth in the upper and middle crust. This body indicated expansion of the upper and middle crust, which may be obstructed by the northeastward extrusion of the Tibetan Plateau, causing the crustal materials to converge and increasing the density of the crust south and northwest of the epicenter as mentioned above.

## Discussion

Firstly, we estimated the contributions from the vertical deformation and hydrology on gravity changes. According to the approximate formula Δ*g = *2π*Gρ*Δ*h*, if Δ*g* = 1 μGal and the crustal density *ρ = *2670 kg/m^3^, the vertical deformation Δ*h* should reach ~9 mm. However, the surface uplift in the NETP was < 6 mm/a revealed by the annually levelling measurements^18,28,30^ or ~1.3 mm/a from the GPS solution^31^. We inferred that the maximum of gravity decrease was < 1 μGal/a, which was too small to account for the observed gravity-change values and could be neglected. Compared with the amplitude of the gravity changes in the NETP, the effects of other factors such as the hydrology were also small^12,28^, which also could be inferred from the groundwater storage change of < 10 mm/a (i.e. < 1 μGal/a on gravity change based on the above approximate formula) from GRACE observations^32^. Therefore, in this study we considered the obtained gravity changes as the DC results in the local crustal materials.

Previous studies suggested the close relationship between gravity changes and earthquakes, and some significant investigations of the subsurface evolution preceding the typical earthquakes^4,5,8,9,12^, and in common, could be explained in terms of the redistribution of the crustal materials^17^, which represented in two forms: negative and positive DCs. The negative DCs were mainly resulted from when crustal materials shifted from their original location and were not replaced or were replaced by a smaller volume of new material; this process may be considered as expansion of the original crustal material. The positive DC was mainly caused by the materials shifting into the area or convergence of the crust due to tectonic forces. Although this work was a preliminary study, our crustal DC results could generally account for the dynamic process preceding the 2016 Menyuan earthquake. The density changes from the inversion results mainly dominated the upper and middle crustal movement around the seismic region (Figs. 2–5) and to some extent showed correlation with the pre-earthquake tectonic process.

**Figure 6:**
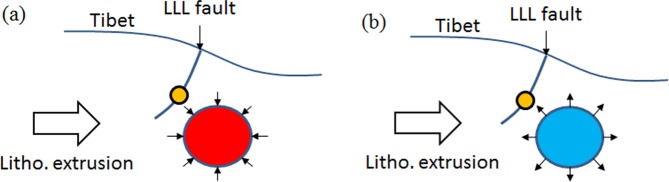
Schematic representation of the crustal density-change process before the 2016 Menyuan earthquake. (**a**) The density of the seismogenic body increased approximately 1.5 years before the earthquake. (**b**) The density of the seismogenic body decreased approximately four months before the earthquake. The orange circle shows the seismic focus of the 2016 Menyuan earthquake.

The NWW–SEE trending strike-slip LLL fault partly accommodated the northward movement of the Tibetan Plateau and allowed for northeastward crustal extrusion^21,23^. The crustal materials with northward extrusion were partly transferred northeastward and southeastward, which should be the major reason of the positive DCs in the southeast segment of the LLL fault and negative DCs in the other regions between the SLM and the DM faults during 2011–2012 (Fig. 2). The crustal DCs should be induced by the interaction between the Tibetan Plateau and adjacent blocks, such as the Alxa block^25^, however, Fig. 6 was not enough evidence to the pre-earthquake activity preceding the 2016 Menyuan earthquake. Regarding the location of the epicenter, some studies argued this event ruptured on the northeastward arc-shaped secondary fault of the LLL fault^22^, which had dip-slip properties, suggested that LLL fault partly absorbed the northeastward movement of the Tibetan Plateau. At this point, we proposed that the crustal positive DCs in the northwest segment of the LLL fault during 2013–2014 and 2014–2015 (Figs. 4 and 5) were caused by the merging of the faults with the Tibetan lithospheric extrusion. The thrusting RM fault south of the Xining Basin restrained the integrative actions of the right-lateral RM fault east of the Xining Basin and the left-lateral DM fault^33^, suggesting the density-change increasing generally. Furthermore, the lower Poisson ratio beneath the Xining Basin and northwest segment of the LLL fault^34^ would increase the rheological properties of the upper and middle crust and the crustal materials were more likely to be redistributed.

Continuous northeastward extrusion of the Tibetan lithosphere^25,35^ would provide the driving force that creates the hotter crust^36^, allowing partly melted materials flow continuously into the NWW–SEE belt region between the DM and the NQM faults, also referred to the seismic region. This agreed with Li and Fu^8^ who proposed that the gravity-change related to the Tangshan earthquake were mainly caused by liquid materials which flowed into the rock pores. When the relatively hot crustal materials of the Tibetan Plateau^36^ flowed into the fractures or pores of the potential seismogenic body, the temperature-change affected the rock porosity and the strain energy release rate^37^. Owing to the resistance of the rigid Alxa and Ordos blocks, the crustal materials should be gathered and the local density should increase. However, the crustal rock DC was negative around the epicenter except in the southeastern segment of the LLL fault during 2011–2012 (Fig. 2), which suggested that the expansion dominated the seismic region with the likely crustal movement direction being southeast. The flow materials filled the potential seismogenic body and raised the rock density (Fig. 3). The rock density increases to a maximum approximately 1.5 years before the earthquake (Fig. 4), which suggested the significant dilatation of the seismogenic body. The continuous crustal flow should result in surface uplift, as reported by Zhang^30^, which should reduce the gravity. Crustal loading and continuous extrusion inhibit the expansion of the middle crust with its relatively high Poisson ratio^34^. Subsequently, the crustal materials presented westward and southward expansion corresponding to the increasing seismic travel time near the epicenter^38^, and convergence beneath the west segment of the LLL fault and the Xining Basin (Fig. 5), suggesting that the diffusion of the seismogenic body was an indication of pre-earthquake activity four months before the earthquake.

Figure 6 showed a schematic representation of the process of crustal DCs before the 2016 Menyuan earthquake. The epicenter was not the site of the maximum of the DC amplitude, but had lower amplitude. The body of increasing density east of the epicenter (Fig. 4) was formed by the partly melted crustal materials flowing into rock fractures or pores. Accordingly, the outflow of the partly melted crustal materials increased the crustal density decrease (Fig. 5). We proposed that the process of convergence and expansion revealed the gradual accumulation of the seismogenic energy; once the rocks of the seismogenic body reached the ultimate strength, the earthquake occurred.

The gravity-change process before the thrust-type 2008 Wenchuan earthquake also presented alternating increasing and decreasing gravity levels near the epicenter^10,12^. However, unlike the 2016 Menyuan earthquake, the Wenchuan earthquake occurred after the gravity increased rather than decreased, which implied that thrust-type earthquakes should rupture during either convergence or expansion of the seismogenic body, depending on the fracture condition of the seismogenic body. The 2016 Menyuan earthquake occurred after the expansion of the seismogenic body inferred from the deceasing density (Fig. 5), suggesting that the processes preceding this earthquake were analogous to the DD model proposed by Nur^9^.

## Conclusions

Changes in the crustal density before the 2016 Menyuan earthquake beneath the NETP were presented and the seismogenic process of the earthquake was investigated based on the derived DC results. An anomalous body with increasing density formed in the upper and middle crust east of the epicenter approximately 1.5 years before the earthquake. Approximately four months before the earthquake, the density of the crustal materials near the epicenter decreased. The seismogenic body underwent a process of convergence and expansion against the background of the Tibetan lithospheric extrusion, which corresponded to the rock transforming from a state of dilatation to diffusion. Our results agreed with the DD model of Nur^9^ for the seismogenic process.

### Data and method

#### Gravity-change data in the NETP

Analysis of the gravity-change data obtained from the gravity network which covers the NETP is an important part of the seismic monitoring program in China. Two types of the periodical repeated gravity observations are carried out by China Earthquake Administration (CEA): absolute gravimetry at the benchmarks and relative gravimetry at every station. The time buckets are from March to May and from July to September every year. More than 100 stations using the FG5(X)/A10 absolute gravimeters within the gravity network in mainland China^39,40^ are available for gravity datums regarding the scale-factor calibration of the relative gravimeters and the classical adjustment. The procedure of round-trip observation is carried out for the relative gravimetry, that is, A → B → C → ··· → C → B → A. Considered the uncertainties of reading values from relative gravimeters (usually 10 μGal) and of absolute observations (5 μGal, including reduction), the Liudong Gravimetry Adjustment (LGADJ) software package is used for net adjustment and to obtain the gravity data at each measured gravity points. During the adjustment calculations, the corrections of earth tide, drift of relative gravimeters and atmospheric pressure are addressed according to the convention^41^. The accuracies of the gravity values at measured points from the net adjustment are almost better than 15 μGals, even better than 10 μGals in most cases, which ensure the reliability of the gravity changes with time at the measured stations. Therefore, gravity-change datasets are regarded as one of the most effective geophysical data for investigating the deep processes before the strong earthquakes^10,12^, including the 2016 Menyuan earthquake^27,28,40^.

**Figure 7:**
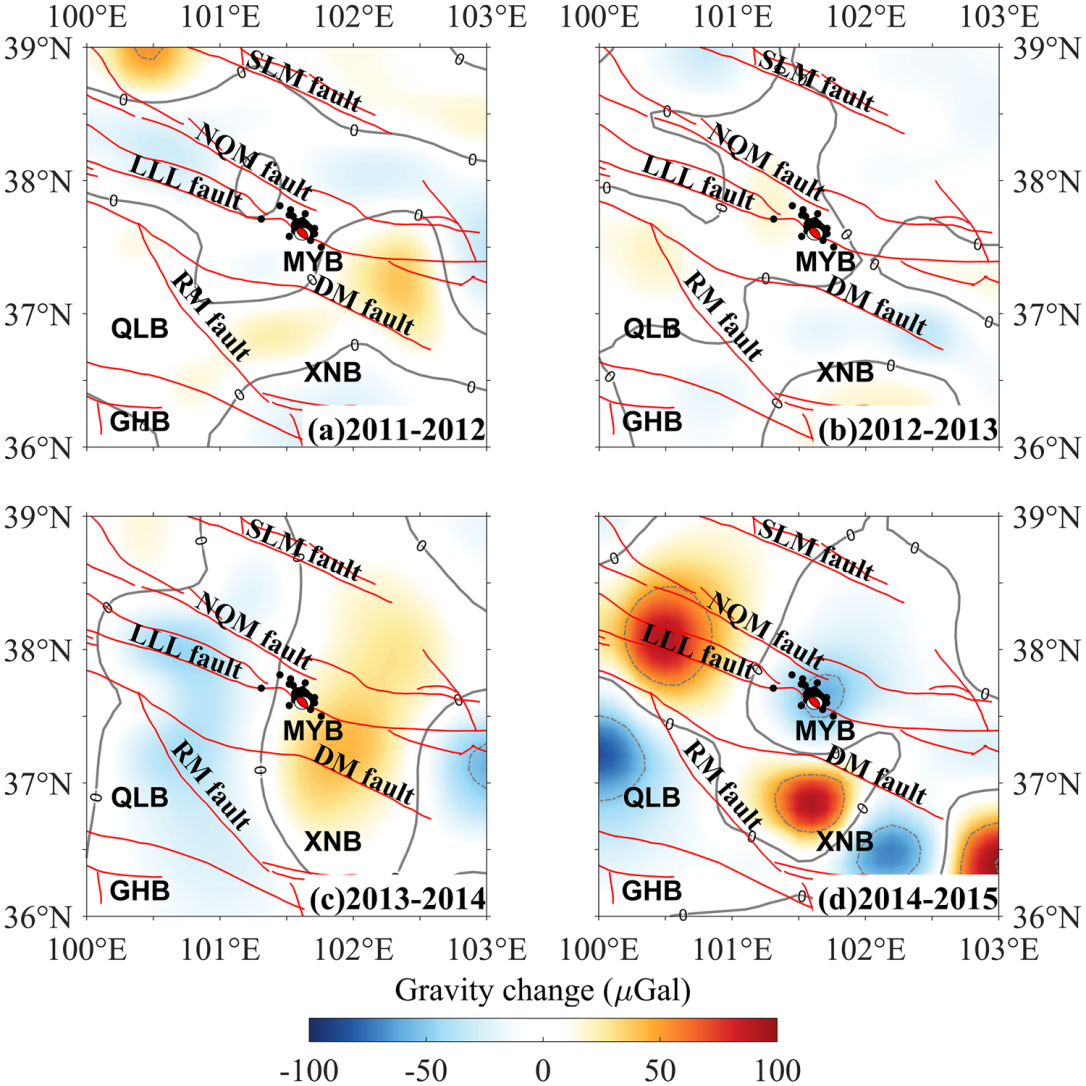
Gravity changes on a one-year timescale from 2011 to 2015. The focal mechanism solutions of the 2016 Menyuan earthquake are from USGS^26^. The abbreviations are the same as those shown in Fig. 2.

Terrestrial gravity data with spatial resolution of approximately 30 km in the NETP were obtained from the Gravity Network Centre of China (GNCC). The data was processed based on the classical adjustment by using LDADJ software to obtain the gravity values for each measurement period. The one-year time-scale data was used here, which may minimize the annual and semi-annual effects, such as hydrological and atmospheric pressure effects, based on the available data. The gravity differences at every station for two consecutive years were obtained by subtracting the gravity values of the previous year from those of one year. For gravity-change data, many of the standard gravity corrections (e.g. Bouguer and terrain corrections) were not required, because the corrections would be identical for the measurements made at each gravity station every year. Figure 7 presented the gravity-change maps on a one-year timescale from 2011 to 2015 before the 2016 Menyuan *M*_S_6.4 earthquake. The gravity-change data used for this study were gridded to 0.1° × 0.1° and first-order details of the discrete wavelet transformation were removed to reduce the effects of the high-frequency noise. Negative gravity-change in the northwest segment of LLL fault (east of 101°E) presented in the periods 2011–2012, 2012–2013 and 2013–2014 (Fig. 7a–c), and then positive gravity-change dominated this region in the period 2014–2015 (Fig. 7d). The epicenter almost located in the region with positive gravity-change during 2012 and 2014 (Fig. 7b,c), subsequently, changed to distinct negative gravity-change in the period 2014–2015 (Fig. 7d). Although the quasi-stable adjustment, rather than the classical adjustment, was applied in several previous studies^27–29^, the major characteristics of the gravity changes were in accordance with ours shown in Fig. 7. Since the gravity changes resulted from preparation of the 2016 Menyuan earthquake had been described in detail^27–29^ and were unnecessary restatement here.

#### Gravity inversion method

The underground domain is divided into *M* prisms, and the gravity effects caused by the DC at the *i*th point, Δ*g*_*i*_, is the sum of the gravity effect of each prism, given by

1 $$\begin{array}{cc}\Delta {g}_{i}=\mathop{\sum }\limits_{j=1}^{M}{a}_{ij}{v}_{j}+{e}_{i} & i=1,\ldots ,N\end{array}$$

where *a*_*ij*_ is the element of the kernel matrix ***A***, *v*_*j*_ indicates the density change of the *j*th prism and *e*_*i*_ is the *i*th element of the noise matrix ***E*** associated with the observation data. The observation equation Eq. (1) can also be given in matrix form:

2 $${\boldsymbol{G}}={\boldsymbol{AV}}+{\boldsymbol{E}}$$

The compact gravity inversion method^42,43^ is the primary algorithm used here to determine the underground density changes. The distribution of the density changes ***V*** is determined to minimize the objective function composed of the density changes underground and errors of the observed gravity changes:

3 $$\mathop{\sum }\limits_{j=1}^{M}{w}_{vj}{v}_{j}^{2}+\mathop{\sum }\limits_{i=1}^{N}{w}_{ei}{e}_{i}^{2}\to \,\min $$

subject to Eq. (2). *w*_*vj*_ and *w*_*ei*_ are weighting functions of the density-change and gravity-change data, respectively. The minimization of the weighted least-squares problem (Eq. (3)) requires that the partial derivative with respect to ***V*** is equal to zero, whereby the solution following Last and Kubik^42^ is given by

4 $${\boldsymbol{V}}={{\boldsymbol{V}}}_{0}+{{\boldsymbol{W}}}_{v}^{-1}{{\boldsymbol{A}}}^{T}{({\boldsymbol{A}}{{\boldsymbol{W}}}_{v}^{-1}{{\boldsymbol{A}}}^{T}+{{\boldsymbol{W}}}_{e}^{-1})}^{-1}{\boldsymbol{G}}$$

where, ***W***_*v*_ and ***W***_*e*_ are the diagonal matrixes composed of *w*_*vj*_ and *w*_*ei*_ in Eq. (3), respectively. It is known that the kernel matrix ***A*** decays with the inverse squared depth. The depth-weighting matrix ***W***_*d*_ is introduced to overcome the density mostly concentrated near the surface^44,45^, and then the solution Eq. (4) can be modified as

5 $${\boldsymbol{V}}={{\boldsymbol{V}}}_{0}+{{\boldsymbol{W}}}_{d}^{-1}{{\boldsymbol{W}}}_{v}^{-1}{{\boldsymbol{W}}}_{d}^{-1}{{\boldsymbol{A}}}^{T}{({\boldsymbol{A}}{{\boldsymbol{W}}}_{d}^{-1}{{\boldsymbol{W}}}_{v}^{-1}{{\boldsymbol{W}}}_{d}^{-1}{{\boldsymbol{A}}}^{T}+{{\boldsymbol{W}}}_{e}^{-1})}^{-1}{\boldsymbol{G}}$$

Combining the constraint matrixes with the minimum volume ***W***_*v*_ and depth-weighting ***W***_*d*_ should speed up the iterative convergence in comparison to using only one of the matrixes. The solution can be obtained directly from Eq. (5) for any initial model ***V***_0_. In this study, the iteration starts from ***W***_*v*_ = ***I*** and ***V***_0_ = 0. The numerical test for the availability of the inversion method can be found in the Supplementary Information. 

## Supplementary information

Numerical test of the inversion method

## Data Availability

The datasets of gravity change used in this study are available from Gravity Network Centre of China (GNCC) on reasonable request.
