# Supplementary material for: Insight into the preparation of the 2016 MS6.4 Menyuan earthquake from terrestrial gravimetry-derived crustal density changes
Source: Sci Rep. 2019 Dec 3;9:18227. doi: 10.1038/s41598-019-54581-5 (PMC6890680; doi:10.1038/s41598-019-54581-5)
Supplement: Supplementary file 1 — Numerical test of the inversion method [file 41598_2019_54581_MOESM1_ESM.docx]

**Insight into the preparation of the 2016 *M*_S_6.4 Menyuan earthquake from terrestrial gravimetry-derived crustal density changes**

Songbai Xuan, Shuanggen Jin, Yong Chen, Jiapei Wang

**Supplementary information**

**Numerical test of the inversion method.** A combination model composed of two prisms with different size, depth and density changes (Fig. S1a) is designed for the numerical tests. Table S1 presents the model parameters and Fig. S1b shows the gravity changes calculated from the designed model. First, the underground domain is divided into 16,810 prisms of dimensions 2.5 × 2.5 × 5 km. The inversion method mentioned above is used to determine the density changes underground. After six iterations, the distribution of the density changes is obtained (Fig. S2). Positive density changes occur at depths of 10–15 km (Fig. S2b) and 15–20 km (Fig. S2c), while negative density changes are found at depths of 20–25 km (Fig. S2d) and 25–30 km (Fig. S2e). The amplitude of the residuals (Fig. S2g) is less than 1 μGal with an average of −0.183 μGal and standard deviation of 0.180 μGal. The gravity response of the inversion results (Figure. S2h) agrees with that of the designed model (Fig. S1b). Therefore, this method can be applied to reliably determine the underground density-change bodies with different horizontal scales and different buried depth.

|  | **Central coordinate**  **(km)** | | | **Size**  **(km)** | | | **Density Change**  **(g/m^3^)** |
| --- | --- | --- | --- | --- | --- | --- | --- |
|  | *x* | *y* | *z* | *x* | *y* | *z* |  |
| Prism 1 | -25 | 0 | 15 | 10 | 10 | 10 | 200 |
| Prism 2 | 25 | 0 | 25 | 20 | 20 | 10 | -200 |

**Table S1.** Statistic information of the testing model.


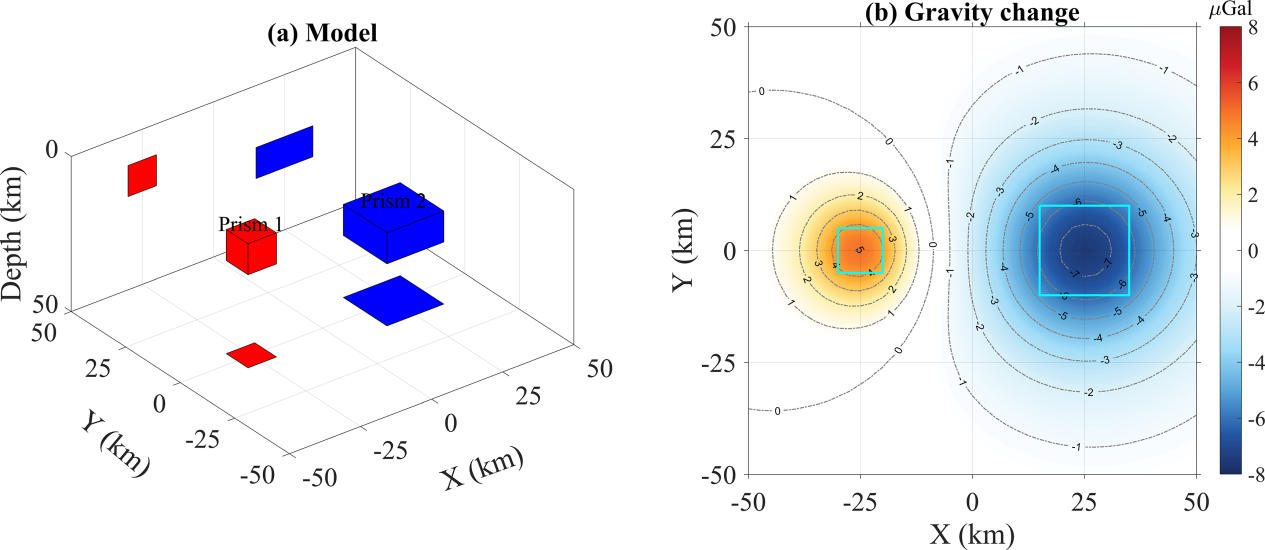


**Figure S1.** (a) Numerical model and (b) its gravity changes. The parameters of the model are presented in Table 1.**
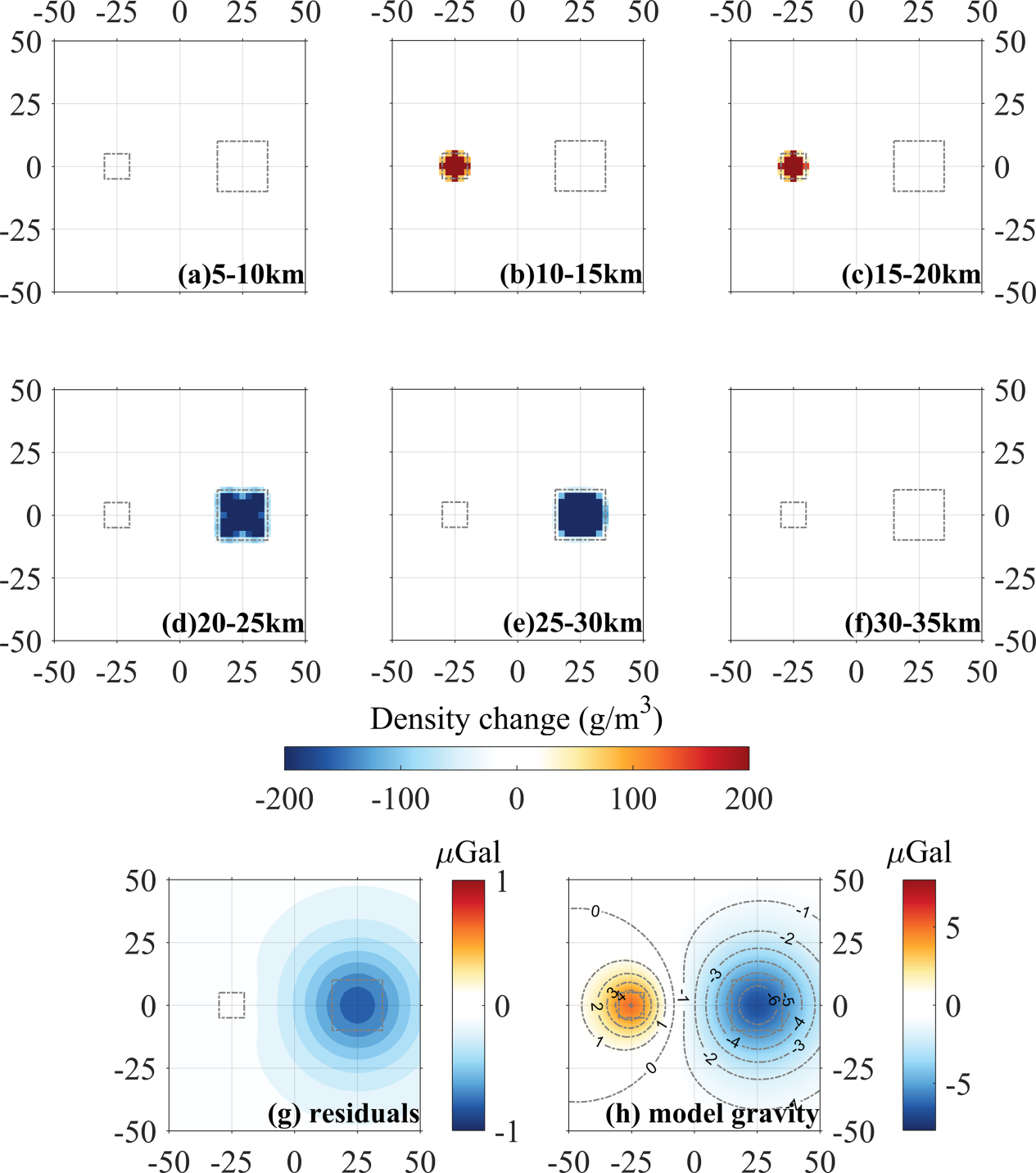
**

**Figure S2.** Inversion result of the model. (a)–(f) Slice maps of density changes from 5–35 km. (g) Residuals map. (h) Gravity response of the inversion result. The grey dotted lines outline test prism 1 and prism 2.
